# Cryptic protein-protein interaction motifs in the cytoplasmic domain of MHCI proteins

**DOI:** 10.1186/s12865-016-0154-z

**Published:** 2016-07-19

**Authors:** Karla K. Frietze, Adlai L. Pappy, Jack W. Melson, Emily E. O’Driscoll, Carolyn M. Tyler, David H. Perlman, Lisa M. Boulanger

**Affiliations:** Department of Molecular Biology, Princeton University, Princeton, NJ 08544 USA; Princeton Neuroscience Institute, Princeton University, Princeton, NJ 08544 USA

**Keywords:** MHC class I, MHCI, PDZ, PDZ ligand, Cytoplasmic, Synapse, Scaffolding, Brain, Immune

## Abstract

**Background:**

Major histocompatibility complex class I (MHCI) proteins present antigenic peptides for immune surveillance and play critical roles in nervous system development and plasticity. Most MHCI are transmembrane proteins. The extracellular domain of MHCI interacts with immunoreceptors, peptides, and co-receptors to mediate immune signaling. While the cytoplasmic domain also plays important roles in endocytic trafficking, cross-presentation of extracellularly derived antigens, and CTL priming, the molecular mediators of cytoplasmic signaling by MHCI remain largely unknown.

**Results:**

Here we show that the cytoplasmic domain of MHCI contains putative protein-protein interaction domains known as PDZ (*P*SD95/*d*isc large/*z*onula occludens-1) ligands. PDZ ligands are motifs that bind to PDZ domains to organize and mediate signaling at cell-cell contacts. PDZ ligands are short, degenerate motifs, and are therefore difficult to identify via sequence homology alone, but several lines of evidence suggest that putative PDZ ligand motifs in MHCI are under positive selective pressure. Putative PDZ ligands are found in all of the 99 MHCI proteins examined from diverse species, and are enriched in the cytoplasmic domain, where PDZ interactions occur. Both the position of the PDZ ligand and the class of ligand motif are conserved across species, as well as among genes within a species. Non-synonymous substitutions, when they occur, frequently preserve the motif. Of the many specific possible PDZ ligand motifs, a handful are strikingly and selectively overrepresented in MHCI’s cytoplasmic domain, but not elsewhere in the same proteins. Putative PDZ ligands in MHCI encompass conserved serine and tyrosine residues that are targets of phosphorylation, a post-translational modification that can regulate PDZ interactions. Finally, proof-of-principle in vitro interaction assays demonstrate that the cytoplasmic domains of particular MHCI proteins can bind directly and specifically to PDZ1 and PDZ4&5 of MAGI-1, and identify a conserved PDZ ligand motif in the classical MHCI H2-K that is required for this interaction.

**Conclusions:**

These results identify cryptic protein interaction motifs in the cytoplasmic domain of MHCI. In so doing, they suggest that the cytoplasmic domain of MHCI could participate in previously unsuspected PDZ mediated protein-protein interactions at neuronal as well as immunological synapses.

**Electronic supplementary material:**

The online version of this article (doi:10.1186/s12865-016-0154-z) contains supplementary material, which is available to authorized users.

## Background

**Fig. 1:**
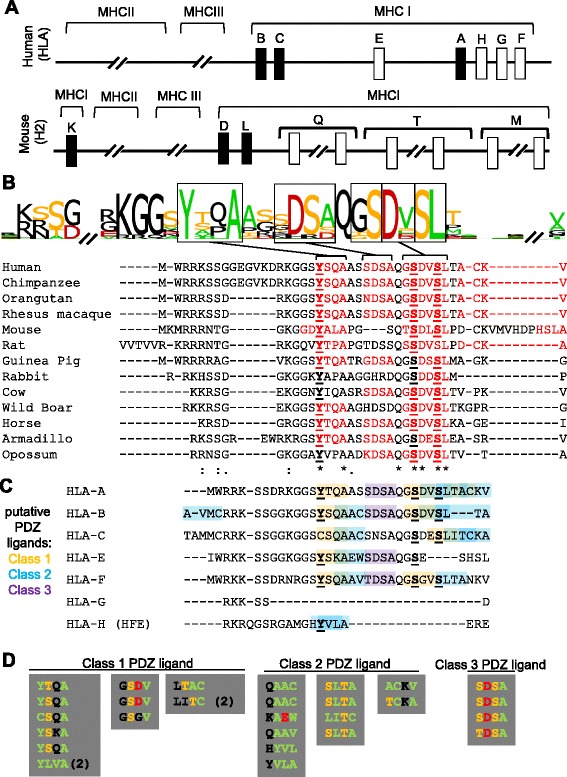
MHCI gene family, and conservation of PDZ ligand motifs in MHCI cytoplasmic domains. **a** Greatly simplified schematic of the MHCI genomic region in humans (HLA, on chromosome 6, *top*) and mice (H2, on chromosome 17, *bottom*), showing the relative positions of the classical (*black boxes*) and non-classical (*white boxes*) MHCI genes or gene families. Multi-member gene clusters are indicated by breaks in the chromosome. Numerous genes and pseudogenes have been omitted for clarity, and distances are not to scale. Centromeric is to the left. Adapted from [62]. **b** Logo showing conservation of amino acids within closest-match homologues of the human classical MHCI HLA-A. The height of each letter corresponds to the extent of conservation across species. Color code: ST, *orange*; YFWCMVILA, *green*; DE, *red*; all others black. Below, individual source sequences. Putative PDZ ligand motifs are in red. Previously noted conserved serine and tyrosine residues that can be phosphorylated in some species (underlined in bold in **b** and **c**; see text) are embedded in PDZ ligand motifs in several MHCI proteins. Similar alignments have been performed previously (e.g., [15]). * = conserved; : = semi-conserved; . = similar. **c** Aligned amino acid sequences of the cytoplasmic domains of human MHCI proteins, with putative PDZ ligand motifs highlighted (class 1PDZ ligand, orange; class 2, blue; class 3, purple). Consensus motifs: class 1 PDZ, S/T-X-Y/F/W/C/M/V/I/L/A; class 2 PDZ, ΦXΦ (Y/F/W/C/M/V/I/L/A-X-Y/F/W/C/M/V/I/L/A); class 3 PDZ, D/E-X-Y/F/W/C/M/V/I/L/A [49]. Human reference sequences obtained from NCBI, alignments performed using T-Coffee [34]. **d** Conservation of PDZ ligands across human HLA genes from **c**, despite non-conservative substitutions. In two cases, a class 1 ligand motif is converted to class 2 by the substitution (2), but remains a putative PDZ ligand. Color code as in (**b**)

The major histocompatibility complex class I (MHCI) is a multi-gene family known for its roles in the immune response. Classical MHCI proteins (HLA-A, −B, and –C in humans; H2-K, −D, and –L in mice; Fig. 1a) present diverse peptides, derived primarily from cytosolic proteins, for recognition by CD8+ cytotoxic T lymphocytes (CTLs). In contrast, non-classical MHCI proteins (HLA-E, −F, and –G in humans; members of the H2-T, −Q, and -M gene clusters in mice) present more specialized peptide or lipid repertoires, or are physically incapable of presenting antigen, and their functions are relatively poorly understood.

In addition to MHCI’s well-known functions in the immune response, recent studies have identified critical, unexpected roles for MHCI in the establishment, function, and modification of neural circuits in the healthy mammalian nervous system (reviewed in [1–4]). MHCI limits synapse density in multiple brain regions [5–9], limits hippocampal synaptic transmission mediated by NMDA-type glutamate receptors (*N*-methyl *D*-aspartate receptors [10]), and is required for normal NMDAR- dependent synaptic plasticity and NMDAR- and hippocampus- dependent forms of learning and memory [11]. Despite the important roles of MHCI in determining neuronal structure and function, the protein interactions that mediate MHCI’s non-immune functions in neurons have not been identified.

MHCI proteins are generally single pass transmembrane proteins comprised of an extracellular domain, a transmembrane domain, and a short (~20–40 amino acid) cytoplasmic domain. The extracellular domain of MHCI binds to peptides, immunoreceptors and coreceptors to participate in immune surveillance. Although the cytoplasmic domain is dispensable for MHCI delivery to the cell surface and for effective presentation of endogenous antigens in vitro [12, 13], subsequent studies showed that the cytoplasmic domain regulates MHCI’s endocytosis and delivery to the endoplasmic reticulum, and is required for cross presentation of extracellularly derived antigens [14–25]. MHCI’s cytoplasmic domain also contains conserved serine and tyrosine residues, some of which are phosphorylated in vitro and in vivo [14, 15, 20, 26–34]. Despite this and other evidence suggesting that the cytoplasmic domain of MHCI is functionally important [17, 35–44], little is known about the sequences or binding motifs that might mediate these functions. As a result, the idea that MHCI might participate in cytoplasmic signaling remains controversial.

Here we identify potential mediators of cytoplasmic signaling by MHCI: cryptic putative protein-protein interaction motifs known as PDZ (*P*SD95, *d*isc large, *z*onula occludens-1) ligands. Multiple bioinformatic analyses suggest that PDZ ligand motifs in the cytoplasmic domain of MHCI are under positive selective pressure, and biochemical assays demonstrate that the cytoplasmic domain of MHCI can bind directly and specifically to PDZ domains in vitro. These results reveal that MHCI proteins may participate in PDZ interactions with transmembrane or cytoplasmic proteins, and suggest a possible mechanism by which MHCI could mediate known and novel functions at both immunological and neuronal synapses.

## Results

### PDZ ligand motifs are found in the cytoplasmic domain of mouse and human MHCI proteins

Previous studies identified conserved tyrosine and serine residues in the cytoplasmic domain of MHCI immune proteins, some of which can be phosphorylated in vivo and in vitro [14, 15, 20, 26–34], but the functional importance of these conserved residues has remained largely unknown. When examining the amino acid sequence of the human classical MHCI HLA-A, we found that these conserved tyrosine and serine residues are embedded in sequences that resemble PDZ ligands (Fig. 1b).

PDZ ligands are short (~4 amino acid) motifs that are recognized by larger (~80–90 amino acid) binding pockets known as PDZ domains. PDZ domains are found in more than 200 proteins in humans, and have been identified in viruses, bacteria, yeast, plants, and animals. PDZ interactions regulate protein localization, binding specificity, and modification (reviewed in [45]), and form the backbone of scaffolds that organize diverse cell-cell contacts, including both immunological and neuronal synapses (e.g., [46], reviewed in [47]).

Most PDZ ligands characterized to date lie at the extreme carboxy terminus (C-terminus) of transmembrane or cytosolic proteins. These C-terminal PDZ ligands have been divided into classes, based on the properties of the residues in specific positions in the ligand motif, although the exact composition and number of consensus motifs is a topic of ongoing research ([48–51]; Additional file 1: Figure S1). In addition to C-terminal ligands, a growing body of evidence supports the idea that PDZ domains can bind to non-C-terminal (internal) ligands. Indeed, internal PDZ ligands may be far more common than previously appreciated [49, 52–61]. Although less well-characterized than C-terminal ligands, internal PDZ ligands might have similar consensus motifs, because consensus properties for both C-terminal and internal ligands will be dictated by the sterics and charge of the PDZ domain binding pocket. In support of this possibility, internal PDZ ligands identified in unbiased, high-throughput yeast two-hybrid screens generally match the requirements for C-terminal PDZ ligands for the same PDZ domains [58].

We observed that the conserved serines in the cytoplasmic domain of HLA-A are embedded in two overlapping putative internal PDZ ligands: GSDV, a class 1 PDZ ligand motif (consensus motif [X S/T X Φ], where X is any residue and Φ is hydrophobic) and DVSL, a class 2 PDZ ligand motif (consensus motif [X Φ X Φ] [49–51]). In addition, the conserved tyrosine is embedded in a class 1 PDZ ligand motif, YTQA. To assess if these motifs are under positive selective pressure, which is a hallmark of functionally relevant motifs, we identified best-match homologues of human HLA-A in other vertebrate species, using the online database search STRING (http://string-db.org/; see Methods). Because the MHCI gene region has undergone extensive duplication and deletion events over the course of vertebrate evolution [62], it is difficult to identify true homologues (genes that descended from a common ancestral DNA sequence) across species. Instead, sequences with the highest amino acid sequence conservation were identified in as many species as possible, and aligned using T-Coffee [63] (http://www.ebi.ac.uk/Tools/msa/tcoffee/). Despite variation in the length of the cytoplasmic domain, significant conservation was seen throughout the aligned sequences. All of the sequences have several basic residues immediately adjacent to the hydrophobic transmembrane region, a common feature of transmembrane proteins. These basic residues may interact with the negatively charged phospholipid head groups at the inner face of the membrane, and help prevent movement of the protein perpendicular to the plane of the membrane [64]. In addition, there was striking conservation of several putative PDZ ligand motifs (Fig. 1b).

HLA-A is one of three classical MHCI genes in the human genome, which also encodes several non-classical MHCIs and MHCI-like proteins. Similarly, in mice, there are three classical MHCI genes and several non-classical MHCI genes (Fig. 1a). To determine if other MHCI proteins also contain potential PDZ ligands, we used a custom built MATLAB program to identify all consensus motifs in the cytoplasmic domain of human and mouse MHCI proteins. Full length amino acid sequences for seven human MHCIs and MHCI-like proteins and 18 mouse MHCI proteins were obtained from NCBI (National Center for Biotechnology Information), and the cytoplasmic domains were identified using splicing information, and aligned within species using T-Coffee [63]. For this analysis, we used a relatively broad categorization scheme to identify potential class 1 [(S/T) X (Y/F/W/C/M/V/I/L/A)], class 2 [(Y/F/W/C/M/V/I/L/A) X (Y/F/W/C/M/V/I/L/A)] and class 3 [(D/E) X (Y/F/W/C/M/V/I/L/A)] PDZ ligands [49].

Several notable patterns emerged. First, the short cytoplasmic domains of nearly all of the MHCI proteins examined contain one or more putative PDZ ligands. Of the seven human sequences examined, all but one (HLA-G, which has the shortest cytoplasmic domain, only six amino acids) contain at least one potential PDZ ligand motif (Fig. 1c). Similarly, 16 of the 18 mouse cytoplasmic sequences examined contain one or more putative PDZ ligand motifs (Additional file 2: Figure S2). Second, PDZ ligand motifs in the cytoplasmic domain of MHCI do not appear to be randomly distributed. Instead, PDZ ligand motifs of a particular class (1, 2, or 3) tend to occur at the same position. For example, five of the six motif-containing human MHCIs contain a class 1 PDZ ligand motif at the same position in the alignment (for example, YTQA in HLA-A), while the sixth MHCI has a class 2 ligand in this position (YVLA in HLA-H; Fig. 1c). Third, the putative PDZ ligands in both human and mouse MHCI encompassed the previously observed conserved serine and tyrosine residues that are targets of phosphorylation (Fig. 1b-c & Additional file 2: Figure S2 [14, 15, 20, 26–34]). Fourth, some PDZ ligand motifs overlapped with each other, either in pairs (for example, HYVLA, which contains the overlapping class 2 PDZ ligand motifs HYVL and YVLA) or in longer chains (for example, DVSLTACKV, which contains three potential class 2 PDZ ligands, DVSL, SLTA, and ACKV). Finally, while the exact amino acid sequence of PDZ ligand motifs in a given position was not perfectly conserved, substitutions that occurred were frequently conservative with respect to the PDZ ligand motif. For example, all six motif-containing human MHCI proteins have a PDZ ligand motif in the position where YTQA is found in HLA-A (Fig. 1c), but only one amino acid in the motif, A, was conserved across all six (Fig. 1d). Such degeneracy is a hallmark of PDZ ligands, and may help explain why these motifs were not identified as conserved in MHCI proteins prior to the current study.

### PDZ ligand motifs are enriched in the cytoplasmic domain of MHCI proteins

One challenge of identifying short, degenerate motifs based on sequence alone is that they are likely to occur at a high frequency by chance. Thus the motifs we identified in the cytoplasmic domain of MHCI may or may not be true PDZ ligands. The fact that they occur in identical positions in multiple genes (Fig. 1b-c & Additional file 2: Figure S2), and are conserved across species, despite amino acid substitutions (Fig. 1d), supports the idea that they have not occurred randomly. However, such apparent conservation could also result from a recently shared precursor and insufficient genetic drift, or could be an allosteric structural requirement for other distant motifs or functions. One prediction is that true PDZ ligands might be enriched in the cytoplasmic domain, where PDZ interactions occur, and where PDZ ligands could therefore be under positive selective pressure. To evaluate this possibility, we used MATLAB to identify all class 1 PDZ ligand motifs in the complete amino acid sequence of 20 mouse MHCI and MHCI-like proteins (see Methods for list), using the relatively restrictive class 1 PDZ ligand consensus motif [X (S/T) X (V/L)] [51]. All values were calculated as frequency of ligands per 100 amino acids, to control for the shorter size of the cytoplasmic domain, and overlapping ligands were counted as separate ligands. To establish a baseline for how often these ligand motifs might arise by chance in a given protein, the full amino acid sequence of each protein was scrambled, and the same analysis was performed.

**Fig. 2:**
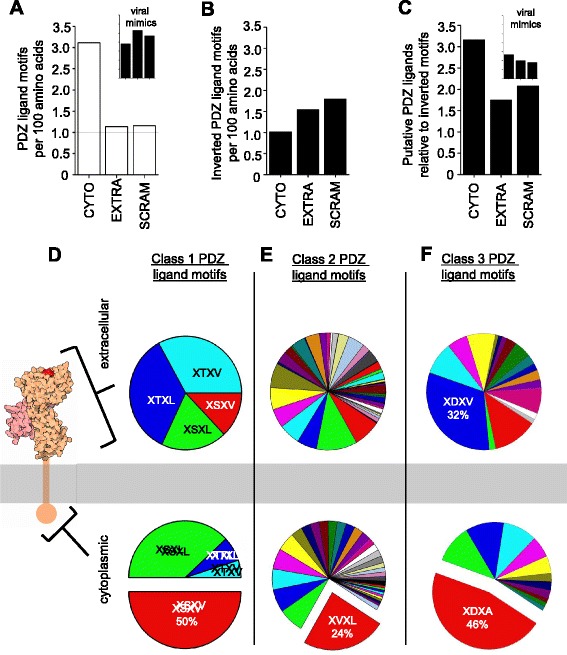
Enrichment of putative PDZ ligand motifs in the cytoplasmic domain of mouse MHCI proteins. **a** Frequency of the class 1 PDZ ligand consensus motif [X (S/T) X (V/L)] [51] in the cytoplasmic domain (“CYTO”), extracellular domain (“EXTRA”) or scrambled full sequence (“SCRAM”) of 20 mouse MHCI and MHCI-like proteins (see Methods for list), represented as motifs per 100 amino acids. PDZ ligand motifs are enriched in the cytoplasmic domain. Inset, frequency of PDZ ligand motifs in five viral MHCI mimics (UL-18, UL-37, UL-142, M144, and M153), which are likely to be under different selective pressures than host MHCI. Axis labels in insets match those in main graph and are omitted for clarity. **b** Frequency of inverted, non-functional class 1 PDZ ligand motifs [(V/L) X (S/T) X] in the same MHCI amino acid sequences. **c** PDZ ligand motifs in MHCI amino acid sequences, represented in relation to the number of inverted motifs in the same domain. Inset, same data from the five viral MHCI mimics used in (**a**). **d** Representation of four specific class 1 PDZ ligand consensus motifs (XSXV, XSXL, XTXV, XTXL) [51] in the extracellular (*top*) versus cytoplasmic (*bottom*) domains of 99 MHCI proteins from 21 species. Serine-containing motifs are significantly over-represented relative to threonine-containing motifs in the cytoplasmic domain. MHCI schematic, rcsb-pdb (http://pdb101.rcsb.org/motm/62). **e**–**f** Representation of specific class 2 (ΦXΦ) and class 3 (X (D/E) X Φ) PDZ ligand motifs, respectively, where Φ = V/I/L/M/F/W/A/C. For a complete list of class 2 and class 3 ligands plotted and their frequencies, see Additional file 3: Figure S3. Color code is the same in both extracellular and cytoplasmic charts

We first compared the frequency of PDZ ligand motifs in the cytoplasmic versus extracellular domains, since PDZ interactions are only known to occur in the cytoplasm. In the extracellular domain, the frequency with which class 1 PDZ ligand motifs occurred was comparable to what was seen in scrambled amino acid sequences (~1 per 100 amino acids), consistent with the idea that PDZ ligands are not selected for in the extracellular space. In contrast, the number of PDZ ligands observed in the cytoplasmic domain was roughly three times this baseline value (Fig. 2a). The significant enrichment of putative PDZ ligands in the cytoplasmic domain of MHCI proteins suggests that there is positive selective pressure on PDZ ligand motifs specifically in the cellular context where PDZ interactions occur.

One unusual set of MHCI-like proteins which may be freed from this selective pressure is viral MHCI mimics. These mimics have been adopted from host genomes, and can allow infected cells to evade NK cell-mediated lysis after viral down regulation of endogenous host MHCI, as part of the viral immuno-evasion strategy [65–67]. Cytoplasmic signaling by these mimics in host cells may not benefit the virus, and therefore may be lost in rapidly evolving viruses. Consistent with this possibility, the cytoplasmic enrichment of PDZ ligand motifs is completely abolished in viral MHCI mimics (Fig. 2a, inset). These results together show that putative PDZ ligands are non-randomly distributed in MHCI proteins, and are specifically enriched in the cellular compartment in which PDZ interactions occur. These findings support the idea that at least some of the PDZ ligand motifs in the cytoplasmic domain of MHCI proteins are functionally relevant.

However, it is possible that the enrichment of these short motifs in the cytoplasmic domain simply reflects a cytoplasmic enrichment of the specific amino acids that make up the consensus motifs, which would increase the probability that they would occur there by chance. To examine this possibility, we searched the same mouse MHCI sequences for the same class 1 PDZ ligand consensus motif [X (S/T) X (V/L)] [51], but inverted the order of residues in the motif (for example, instead of searching for XSXV, we searched for VXSX). Importantly, inverted PDZ ligand motifs contain all of the same amino acids as actual motifs, but are structurally unsuited to bind in PDZ domain binding pockets.

Unlike true PDZ ligand motif sequences, inverted PDZ ligand motifs were not enriched in the cytoplasmic domain (Fig. 2b). Indeed, inverted motifs were slightly less common in the cytoplasmic domain than in the extracellular domain or in scrambled whole sequences. This may be because in the cytoplasmic domain, the relevant amino acids were often part of true PDZ ligand motifs. To quantitatively assess the relative distribution of forward versus inverted motifs, we calculated the ratio of their occurrence in the cytoplasmic and extracellular domains, as well as in scrambled sequences. In the cytoplasmic domain, where PDZ interactions can occur, putative PDZ ligand motifs are more than three times more common than inverted motifs (Fig. 2c). In contrast, the ratio of forward to reverse motifs was comparable in the extracellular domain and in scrambled sequences. The enrichment of forward over reverse motifs is also lost in the cytoplasmic domain of viral MHCI mimics (Fig. 2c, inset). Thus the enrichment of PDZ ligands in the cytoplasmic domain (Fig. 2a) is not simply a consequence of more of the necessary residues being present in this compartment. Instead, PDZ ligand motifs in the cytoplasmic domain of MHCI proteins appear to be under strong positive selective pressure.

### Specific PDZ ligand motifs are preferentially expressed in the cytoplasmic domain of MHCI proteins

The preceding analyses made use of pooled data for broadly defined putative class 1 PDZ ligand motifs [X (S/T) X (V/L)] [51]. To obtain a more detailed picture of the pressures on PDZ ligand sequences in the MHCI cytoplasmic domain, we separately assessed the frequency of occurrence of the specific class 1 PDZ ligand motifs XSXV, XSXL, XTXV, and XTXL, which should each represent roughly 25 % of the class 1 PDZ ligand motifs, if they occur at random. To evaluate PDZ ligand motif distribution in a larger dataset, we obtained full length amino acid sequences for 99 MHCI or MHCI-like proteins from 21 species, using the online database STRING (http://string-db.org/) (see Methods). A custom MATLAB program was used to identify specific putative class 1 PDZ ligands in the cytoplasmic domains of these 99 MHCI proteins, as was done for mouse MHCIs above.

All four class 1 PDZ ligand motifs were detected in both domains of the MHCI proteins examined. However, their relative frequencies differed widely in the two compartments. In the extracellular domain, serine-containing motifs represented 33 % of the total, and each threonine-containing motif occurred at a nearly identical frequency (XTXL, 34 %; XTXV, 33 %). In the cytoplasmic domain, however, there was a striking skewing of the distribution (Fig. 2d). Serine-containing motifs account for 88 % of the total motifs in the cytoplasmic domain, while threonine-containing class 1 PDZ ligands were rarely seen (XTXL, 7 % of the total; XTXV, 5 %). Thus across many species, there is a strong overrepresentation of serine-containing class 1 PDZ ligands, and under-representation of threonine-containing motifs, in the cytoplasmic domain but not in the extracellular domain, further supporting the idea that the PDZ ligand motifs in the cytoplasmic domain of MHCI are under positive selective pressure.

A similar analysis was performed for class 2 PDZ ligand motifs, using the consensus definition XΦXΦ, where Φ = primarily hydrophobic residues (V, I, L, M, F, W, or C) [51]. Likely because this highly degenerate motif can be expressed as a very large number of specific variants, some of the possible variants were never observed in the small cytoplasmic domains. As with class 1 PDZ ligand motifs, class 2 motifs in the extracellular domain occurred at relatively random frequencies, without a clear bias for any one motif. In striking contrast, a single motif, XVXL, comprised nearly a quarter of the total class 2 PDZ ligand motifs observed in the cytoplasmic domain (Fig. 2e). This enrichment is particularly remarkable given the large number of possible class 2 PDZ ligands: XVXL represents only 2 % of the 49 possible degenerate class 2 PDZ ligand motifs [X (VILMFWC) X (VILMFWC)], but is more than ten times more common than that in the cytoplasmic domain in this large, multi-species pool of MHCI proteins. Similar analysis of class 3 PDZ ligand motifs [X (D/E) X (VILMFWC)] also showed striking enrichment of one motif, XDXA, in the cytoplasmic domain (Fig. 2f; for a complete list of class 2 and 3 ligands plotted and their frequencies, see Additional file 3: Figure S3). Unlike class 1 and 2 ligands, however, class 3 ligands also showed a bias in favor of a particular ligand in the extracellular domain, although it was not the same motif that was enriched in the cytoplasmic domain, and its enrichment was not as marked (XDXV represented 32 % of the class 3 ligands in the extracellular domain, as compared to XDXA’s 46 % in the cytoplasmic domain). This may reflect pressure to preserve extracellular XDXV sequences as part of a common non-PDZ motif or structural element. Overall, these results suggest that specific PDZ ligand motifs are under strong positive selective pressure in the cytoplasmic domain of MHCI proteins.

### PDZ ligand motifs in the classical MHCI H2-K^b^ largely fall within single exons

**Fig. 3:**
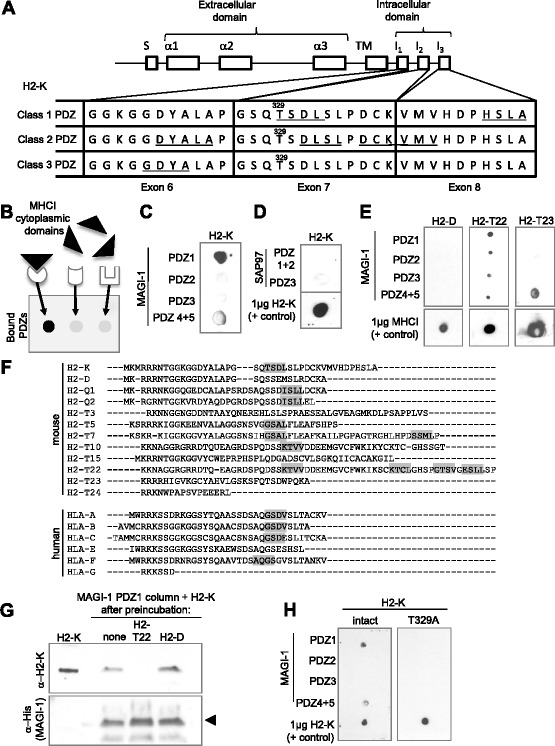
MHCI can bind directly to PDZ domains in vitro. **a** Schematic showing the exons that encode the classical MHCI H2-K^b^. A signal sequence (S) is followed by exons encoding the three extracellular alpha domains, a single exon encoding the transmembrane domain, and three exons (6, 7, and 8) encoding the intracellular domain (not drawn to scale). Bottom, amino acid sequence of exons 6, 7, and 8 in H2-K^b^. Putative PDZ ligand motifs are underlined. Exon 6 encodes the overlapping ligand motifs GDYA, DYAL, and YALA, exon 7 encodes the overlapping ligand motifs TSDL and DLSL, and exon 8 encodes the C-terminal motif HSLA. Only the overlapping motifs DCKV and KVMV span an exon/intron boundary. Notably, KVMV is also among the least conserved putative PDZ ligand motifs in H2-K^b^, since it is not detected in this position in any other mouse MHCI. Intronic structure, NCBI. Adapted from [15]. **b** Schematic of in vitro binding assay. Different recombinant PDZ domain peptides are bound to each membrane spot, and the membrane is panned with GST-tagged recombinant cytoplasmic domains from specific MHCI proteins. A direct interaction between the cytoplasmic domain of MHCI and a given PDZ domain will be apparent as a black spot after visualization of bound anti-GST antibodies. **c** The cytoplasmic domain of the classical MHCI H2-K^b^ can bind directly to PDZ1 and to a lesser extent PDZ4+5 of MAGI-1, but not to PDZs 2 or 3 of the same protein. See Additional file 4: Figure S4B&D for validation of the identity of H2-K peptides, and S4E-F for titration of H2-K^b^ binding. **d** The cytoplasmic domain of the classical MHCI H2-K^b^ does not bind to PDZ1+2 (expressed together) or PDZ 3 of SAP97. **e** MAGI-1 binding by recombinant cytoplasmic domains derived from different classical and nonclassical MHCI proteins. Notably, the ability to bind to PDZ1 of MAGI-1 correlates with the presence of a class 1 PDZ ligand motif, which is present in H2-K^b^ and H2-T22, but not H2-D or H2-T23 (see **f**). **f** Amino acid sequences of the cytoplasmic domains of mouse and human MHCIs, aligned in ClustalW. Highlighted, putative class 1 PDZ ligand motifs (consensus [X – S/T – X – V/L]) that match MAGI-1 PDZ1’s binding preferences [80–82]. **g** Competition assays show that preincubation of column-bound MAGI-1 PDZ1 with cytoplasmic peptides derived from H2-T22, which can bind, but not H2-D, which cannot, precludes subsequent binding by peptides derived from H2-K. *Left*, pure H2-K not applied to a column; “none”, preincubation in buffer alone. H2-K was detected using an antibody against the cytoplasmic domain of H2-K (see Methods). *Bottom*, anti-His antibody shows eluted MAGI-1 peptide (*arrow*). **h** A point mutation in the lone class 1 PDZ ligand motif, TSDL, in the cytoplasmic domain of H2-K^b^ attenuates binding to MAGI-1 PDZ1. The location of the mutated residue (T329) is shown in (**a**)

The cytoplasmic domain of MHCI proteins is relatively short, ~25–40 amino acids in most cases. Despite this, the cytoplasmic domain is encoded in 2–4 separate exons, depending on the particular MHCI gene (exons 6, 7, and 8 in the classical MHCI H2-K^b^; Fig. 3a). While the reason for this fine division of the transcript remains unknown, exons can in some cases represent distinct functional modules [68]. Overlaying splicing information from NCBI with putative PDZ ligand motifs revealed that most of the motifs in H2-K^b^ are encoded in single exons. Exon 6 encodes the overlapping ligand motifs GDYA, DYAL, and YALA, exon 7 encodes the overlapping ligand motifs TSDL and DLSL, and exon 8 encodes the C-terminal motif HSLA. Only the overlapping motifs DCKV and KVMV span an exon/intron boundary. Notably, KVMV is also among the least conserved putative PDZ ligand motifs in H2-K^b^, and is not detected in this position in any other mouse MHCI examined (Additional file 2: Figure S2).

### Proof of principle: the cytoplasmic domains of MHCI proteins can bind directly to recombinant PDZ domains in vitro

The above analyses of amino acid sequence data suggest that PDZ ligand motifs in the cytoplasmic domain of MHCI proteins may be functionally important. To directly test if the cytoplasmic domain of MHCI can engage in PDZ interactions, we asked if recombinant MHCI cytoplasmic domain peptides can bind to recombinant PDZ domain peptides. Mouse H2-K^b^ was cloned from C57BL/6 mouse hippocampus using the primers in Additional file 4: Figure S4A. The cloned sequence was verified by comparing to NCBI reference sequences before insertion into the pGTK vector. We then generated a GST fusion protein with the last 42 amino acids of H2-K^b^ (see Methods). This peptide comprises the complete cytoplasmic domain of H2-K^b^. Three different lines of evidence were used to verify the identity of the recombinant protein product. First, plasmids were subjected to commercial sequencing. The returned sequences were 100 % identical to the endogenous sequence for H2-K^b^, confirming that a correct insert was introduced for in-frame expression of GST-tagged H2-K^b^ cytoplasmic domain. Second, we performed mass spectrometry on samples that contained either intact or mutated GST-H2-K^b^ fusion peptides. These assays identified high levels of recombinant H2-K^b^ cytoplasmic domain peptides, and similarly high levels of the associated GST tag. As expected, H2-K^b^ cytoplasmic domain was not detected in vector-only controls, and peptides with point mutations (see below) differed from intact H2-K^b^ peptide only at the mutated residue. Diagnostic spectra are shown in Additional file 4: Figure S4B-C. This peptide sequence data indicates that the encoded peptides are correctly expressed. Third, purified recombinant MHCI cytoplasmic domain peptides were subjected to SDS-PAGE and western blotting using an antibody raised against the final exon (exon 8) in the cytoplasmic domain of mouse H2-K^b^. A band of the appropriate size is detected in Western blots (Additional file 4: Figure S4D). Thus three independent measures all support the conclusion that the recombinant peptide we produce is a GST-tagged version of the cytoplasmic domain of the classical MHCI H2-K^b^. This GST fusion protein was used to screen PDZ domains that were expressed as His-tagged fusion proteins, purified, and spotted in equal amounts onto nylon membranes. PDZ domains are discrete structural units that can fold properly when expressed as isolated peptides, and display native binding preferences in in vitro binding assays [69–72]. Therefore this type of biochemical array has been used in previous studies to identify proteins that can bind directly to a variety of PDZ domains (e.g., [70]).

In a proof-of-principle analysis, we probed the binding of recombinant, MHCI-derived peptides to PDZ domains from two proteins: MAGI-1 (Membrane-associated guanylate kinase, WW and PDZ domain-containing protein 1), a multi-PDZ domain synaptic scaffolding protein that interacts with NMDA receptors and is required for specific aspects of associative learning and memory [73–75], and SAP-97 (synapse-associated protein 97, also known as DLG1), which regulates NMDAR trafficking [76–79]. These PDZ domains were of particular interest because MHCI regulates NMDA receptor-mediated synaptic transmission and plasticity [10, 11], but the mechanisms by which MHCI regulates NMDARs remain unknown. Membranes were spotted with recombinant PDZ domains and then incubated with recombinant GST-tagged MHCI cytoplasmic domains (Fig. 3b). After extensive washing, robust detection of GST revealed that the cytoplasmic domain of H2-K^b^ bound directly and specifically to PDZ1 of MAGI-1, and less intensely to PDZ4+5 (Fig. 3c), but not to any PDZ domain of SAP97 (Fig. 3d). The specificity of the interaction for only certain PDZ domains suggests it does not simply reflect nonspecific binding between recombinant peptides. The specificity of the interaction is also supported by two other observations: (1) the interaction is absent in GST-only (no MHCI C-tail) controls, and (2) the interaction declines in direct relation to the concentration of MHCI cytoplasmic domain peptides present (Additional file 4: Figure S4E-F). In addition, a single point mutation in the MHCI peptide is sufficient to abolish binding (see below), further supporting the idea that these interactions are not a consequence of nonspecific binding, but represent a real and specific interaction between the cytoplasmic domain of MHCI and particular PDZ domains.

MAGI-1 PDZ1 binds to class 1 PDZ ligands in other proteins which conform to the consensus motif [X –S/T – X –V/L] [80–82]. The cytoplasmic domain of the classical MHCI H2-K^b^, which we find can bind directly to PDZ1 of MAGI-1, contains a single sequence that conforms to this class 1 PDZ ligand motif: TSDL. Several other MHCI proteins contain sequences that conform to this motif in an identical or similar position in amino acid sequence alignments (Fig. 3f). For example, the nonclassical MHCI H2-T22 contains the putative class 1 PDZ ligand KTVV, and we find that H2-T22, like H2-K^b^, can bind directly to MAGI-1 PDZ1, but not PDZ 2 or 3, in vitro (Fig. 3e). In contrast, the closely-related MHCI H2-T23, which lacks a class 1 PDZ ligand motif (Fig. 3f), also fails to bind MAGI-1 PDZ1 in our in vitro binding assay (Fig. 3e). Similarly, the other classical MHCI found in C57BL/6 mice, H2-D, does not contain a putative PDZ ligand that fits this motif (Fig. 3f), and does not appear to bind significantly to MAGI-1 (Fig. 3g). Thus the presence of a class 1 PDZ ligand correlates with the ability of particular MHCI cytoplasmic domains to bind directly and specifically to PDZ1 of MAGI-1.

One prediction, if H2-K and H2-T22 both bind directly and specifically to PDZ1 of MAGI-1, is that binding of one MHCI to MAGI-1 should be competed away by preincubation with the other. To test this prediction, MAGI-1 PDZ1 peptides were bound to a column, and preincubated with cytoplasmic domain peptides from either H2-T22, which binds to MAGI-1 PDZ1, or H2-D, which does not (Fig. 3e). Other columns were preincubated in buffer, as a control. After extensive washing (see Methods), the same columns were incubated in H2-K-derived cytoplasmic domain peptides. Preincubation with H2-T22, but not H2-D, completely abolished H2-K’s ability to bind to MAGI-1 PDZ1 (Fig. 3g). This result suggests that the cytoplasmic domains of H2-K and H2-T22 are competing for occupation of the same site on MAGI-1 PDZ1, as would be expected for a classical PDZ interaction.

To directly test if the class 1 PDZ ligand motif TSDL is necessary for binding to MAGI-1, we eliminated this motif in H2-K^b^, by mutating a key residue in the motif (T329A; TSDL->ASDL). Strikingly, this single point mutation is sufficient to disrupt the direct interaction between the cytoplasmic domain of H2-K^b^ and PDZ1 and PDZ4&5 of MAGI-1 in vitro (Fig. 3h). Together, these results provide the first evidence that the cytoplasmic domain of MHCI can bind directly and specifically to PDZ domains.

## Discussion

Here we show, for the first time, that the cytoplasmic domain of MHCI contains conserved PDZ ligand motifs and can engage in PDZ interactions in vitro. Several lines of evidence suggest that PDZ ligand motifs in the cytoplasmic domain of MHCI are under positive selective pressure. The PDZ ligand motifs are conserved across species, with occasional non-synonymous mutations that still preserve the motif, and are found in consistent positions in the sequences of multiple members of the large MHCI gene family in diverse mammalian species. These motifs are enriched in the cytoplasmic domain, where PDZ interactions occur, despite the lack of amino acid bias to explain this enrichment, and tend to be encoded in single exons. The cytoplasmic enrichment of PDZ ligand motifs is lost in viral MHCI mimics, which evolve rapidly and may no longer be under selective pressure to perform cell-autonomous signaling in host cells. Specific motifs are significantly overrepresented in the cytoplasmic domain, in contrast to the extracellular domain, where the possible motifs appear to occur more randomly. Finally, proof-of-concept experiments demonstrate that the cytoplasmic domain of MHCI can bind directly and specifically to PDZ1 and PDZ4&5 of MAGI-1 in vitro, an interaction that requires a conserved class 1 PDZ ligand that is specifically found in MHCIs that can bind to MAGI-1. Together, these results suggest that MHCI may participate in previously unsuspected cytoplasmic signaling mediated by PDZ interactions.

Putative PDZ ligand motifs are conserved in MHCI proteins from 13 species (Fig. 1b). The position of the motifs, their class, and even their exact amino acid sequence are well conserved. This conservation is particularly remarkable given that exact orthologues of most MHCI genes are difficult to determine in other species, due to frequent addition and loss of genes [62]. Thus the conservation of PDZ ligand motifs noted here, while striking, is still likely an underestimate of the actual conservation across true orthologues. In addition, the MHCI region exhibits a relatively high rate of genetic change over evolutionary time, and overall amino acid sequence conservation is low among MHCI genes from different species. In the face of this high genetic variability, it appears that strong evolutionary pressures have actively conserved PDZ ligand motifs in the cytoplasmic domain of MHCI proteins.

Conservation of PDZ ligand motifs among genes within a species is also striking. Human HLA-A, −B, −C, −D, and –F all show similar patterns of ligand motifs (Fig. 1c). In mouse, where the MHCI gene family is larger, there appeared to be groups of genes that form clusters of conservation (Additional file 2: Figure S2; for example, H2-K, −D, −L, −Q1, and –Q2 form one cluster, H2-T3-7; H2-T9-10 form another). These may reflect a common origin for members of that cluster, and/or functional specialization of certain sets of genes. Non-synonymous substitutions that preserve PDZ ligands in specific positions (see examples in Fig. 1d & Additional file 2: Figure S2) may influence the specificity or dynamics of interactions at that site, while still preserving their ability to engage in PDZ interactions.

The bulk of the PDZ ligands identified to date are in the extreme carboxy-terminus of transmembrane or cytosolic proteins, in part because the C-terminus has been considered part of the consensus in most searches to identify new ligands. However, a growing body of evidence supports the idea that PDZ domains also commonly bind internal ligands [49, 52–61]. Studies comparing internal and C-terminal ligand preferences of specific PDZ domains using high-throughput methods suggest that C-terminal and internal ligands share key features [58], likely because they must both conform to the constraints of the same PDZ binding pocket. Thus current definitions of PDZ ligand motifs, while derived primarily from known and predicted C-terminal ligands, are a valid starting point from which to identify potential novel internal PDZ ligands.

PDZ ligand motifs are defined by short, degenerate consensus sequences, and therefore are likely to occur by chance. This property of ligands may make it relatively easy for new PDZ interactions to arise during evolution, but poses a significant barrier to identifying new internal ligands using sequence information alone. In this study, several independent analyses were used to assess the probability that PDZ ligands in the MHCI cytoplasmic domain arose by chance. These analyses consistently support the idea that at least some of the PDZ ligands in the cytoplasmic domain of MHCI are under positive selective pressure. The degeneracy of PDZ ligand motifs, and the assumption that they are exclusively C-terminal, as well as the relatively recent discovery of PDZ interactions [83], all likely contributed to these important motifs in MHCI not being identified previously. A previous study noted a structural feature of unknown importance that is shared by proteins that undergo endocytosis via coated pits, including HLA-A, which they defined as [(aromatic: F, W, G or Y) (polar: Q, S, or T) (charged: D, E, K, R, or S) (hydrophobic: V, I, L, M, P, or T) (not hydrophobic)] [18]. Importantly, this structural feature encompasses one of the most highly-conserved PDZ ligand motifs (GSDV) we identified in HLA-A (Fig. 1b).

Several independent lines of evidence suggest that the direct interactions between the cytoplasmic domain of the classical MHCI H2-K and PDZ1 of MAGI-1 we report here are not a consequence of nonspecific binding, but represent a real and specific interaction between the cytoplasmic domain of MHCI and particular PDZ proteins. H2-K does not bind to all of the eight PDZ domains from two different proteins tested, but rather binds specifically to PDZ1 and PDZ4+5 of MAGI-1. The H2-K cytoplasmic domains are GST-tagged for detection and purification purposes, but GST alone does not bind to any of the PDZ domains tested, suggesting that any binding is due to residues in H2-K. Binding is concentration-dependent with respect to the level of H2-K present, and can be competed away by preincubation of PDZ1 of MAGI-1 with peptides derived from another MHCI that binds to MAGI-1 in vitro. Finally, binding to MAGI-1 PDZ1 correlates with the presence of a class 1 PDZ ligand, and a single point mutation that disrupts this motif also abolishes H2-K’s ability to bind to MAGI-1 PDZ1. Notably, the fact that a lone point mutation abolishes binding serves as a more powerful negative control for nonspecific binding than either a scrambled or irrelevant peptide, because it differs at only a single amino acid. Together, these results strongly support the idea that the cytoplasmic domain of H2-K, and likely other MHCI proteins including H2-T22, can participate in direct, specific PDZ interactions.

The cytoplasmic domain of MHCI is essential for normal endocytic trafficking of MHCI and subsequent cross presentation of extracellularly derived antigens [14–24]. In particular, a putative tyrosine-based endocytic motif, YXXA, has been implicated in the control of MHCI endocytic trafficking [14, 15, 25]. Of note, YXXA is a class 1 or class 2 PDZ ligand motif in most MHCIs (Fig. 1b-d and Additional file 2: Figure S2). The cytoplasmic domain forms a macromolecular complex with the vesicular trafficking protein Sec23a in the trans-golgi network [17], and may interact with cytoskeletal proteins [41–43]. Several studies over the past three decades have suggested that MHCI may mediate reverse signaling [35–40], but cytoplasmic signaling by MHCI remains controversial, in part because the molecular mediators are largely unknown. The current results raise the possibility that these and other functions of MHCI involve PDZ interactions with transmembrane or cytoplasmic proteins. The cytoplasmic domain of MHCI is phosphorylated in vitro and in vivo at multiple sites, including Y320 of HLA-A and S335 of HLA-B [20, 26, 27, 29, 30], sites that we find are embedded in PDZ ligand motifs (Fig. 1b-c and Additional file 2: Figure S2). It will be of interest to determine if phosphorylation of these residues in the MHCI cytoplasmic domain modifies their ability to participate in PDZ interactions, as has been seen with other PDZ ligands ([84]; reviewed in [85]).

The intron/exon structure of the MHCI cytoplasmic domain is notable: in H2-K^b^, three micro-exons (exons 6, 7, and 8) together encode a polypeptide segment of only ~40 amino acids [86, 87], hinting that the cytoplasmic domain of MHCI may be composed of distinct structural and/or functional modules. Consistent with this possibility, we find that PDZ ligand motifs in the cytoplasmic domain largely fall within single exons (Fig. 3a). There are at least 12 splice variants of the mouse classical MHCI H2-K deposited in NCBI to date, and the cytoplasmic domain is alternatively spliced in all of them. Furthermore, alternative splicing that removes exon 7, the site of a highly-conserved PDZ ligand motif (Figs. 1b and 3), has been observed in many species, including humans [16, 28, 88–90]. Loss of exon 7 is associated with enhanced anti-viral CTL responses in vivo [15], and is associated with slower internalization of MHCI from the cell surface [18]. The current results suggest that alternative splicing could potentially regulate MHCI’s ability to participate in PDZ interactions, for example, in specific tissues and developmental stages or in disease states.

One key feature of internal PDZ ligands is that they must adopt an appropriate conformation to fit into the pocket of the PDZ domain binding groove. Some internal ligands are deformed by interaction with the PDZ domain pocket, allowing the peptide to extend past the end of the pocket [60]. Others form a β-finger, which acts as a pseudo-C-terminus [59, 91, 92]. The cytoplasmic domain of MHCI is predicted to be unstructured, but could potentially form a β-finger through interaction with other proteins. Consistent with this possibility, crystal structures show that the cytoplasmic domain of MHCI is deformed into a tight turn by the HIV protein Nef [93]. Palmitoylation of MHCI [94] may also introduce a kink in the cytoplasmic domain, potentially facilitating interactions with PDZ domain pockets. The conservation of sequences immediately flanking the PDZ ligand motifs in MHCI (Fig. 1b) may reflect an important role for these flanking residues in ensuring that PDZ ligand motifs are presented in the appropriate context for recognition by PDZ domains.

How could PDZ interactions contribute to MHCI function in vivo? Several theories remain to be explored. PDZ interactions could contribute to the trafficking and/or clustering of MHC proteins, or could transduce signals from the extracellular domain. MHCIs are among the most polymorphic genes in the human genome, with thousands of variants sequenced to date. In-depth future studies will be needed to fully profile the binding of all of the different MHCI proteins and variants to the hundreds of different PDZ domains that have been identified in mammals, to determine if the identified interactions are of sufficient strength and specificity to occur in cells, and to characterize the functional significance of these interactions in specific biological contexts. The relative invariance of the cytoplasmic domain across human HLA variants means it is particularly well-suited to mediating aspects of MHC function where allelic variability is less beneficial, for example, in organization of the immunological synapse, or in the non-immune functions of MHCI in the brain (reviewed in [1–4]). PDZ interactions are common at cell-cell junctions, including neuronal and immunological synapses. The current results suggest that MHCI-mediated PDZ interactions could play a central role in immune signaling, and could also contribute to the newly discovered functions of MHCI in the healthy developing and adult nervous system.

## Conclusions

These results identify cryptic PDZ-based protein interaction motifs in the cytoplasmic domain of MHCI. Multiple bioinformatic analyses suggest that PDZ ligand motifs in the cytoplasmic domain of MHCI are under positive selective pressure, and biochemical assays demonstrate that the cytoplasmic domain of MHCI can bind directly and specifically to PDZ domains in vitro. These results identify a mechanism by which MHCI proteins may bind directly to transmembrane or cytoplasmic proteins, and suggest that MHCI could mediate previously unsuspected PDZ signaling at neuronal as well as immunological synapses.

## Methods

### Amino acid sequences

Mouse and human MHCI amino acid sequences were obtained from the National Center for Biotechnology Information (NCBI) Protein Database (http://www.ncbi.nlm.nih.gov/protein). For human MHCI (HLA) genes, for which thousands of variants have been sequenced to date, the reference allele was used (https://www.ebi.ac.uk/ipd/imgt/hla/). Mouse MHCI (H2) sequences were all from the b haplotype, except H2-L, which is not present in the b haplotype, and therefore the d haplotype allele was used.

To obtain full length amino acid sequences for MHCI proteins from other species, STRING version 10 was used (http://string-db.org/) [95]. Entering a given mouse or human MHCI gene name, and using the “Occurrence” function, yielded complete amino acid sequences for the closest-match homologues of that gene in other species. A series of 14 distinct mouse and human MHCI and MHCI-like proteins were searched in STRING (mouse H2-K, −D, −T5, −M5, −T9, FcRn, and HFE; human HLA-A, −B, −C, −E, −G,–H (HFE), and FcRn), and all redundant sequences (those identified in more than one search) were discarded to avoid oversampling bias. This strategy produced a set of 99 non-redundant MHCI amino acid sequences from 21 species. Other searches were performed, but not included, because all products of the search were redundant with those identified in previous searches. Amino acid sequences for viral MHCI mimics (UL-18, UL-37, UL-142, M144, and M153) were also obtained from NCBI.

The location of the cytoplasmic domain of each amino acid sequence was identified by splicing information, hydrophobicity, or alignment with other sequences, or predicted using TOPCONS (http://topcons.cbr.su.se/) and manually curated. To randomize full amino acid sequences of mouse MHCIs for Fig. 2a-c, a free online text scrambler (http://textmechanic.com/Word-Scrambler.html) was used.

### Multiple sequence alignments

Full sequences were aligned using ClustalOmega (http://www.ebi.ac.uk/Tools/msa/clustalo/) [96], and cytoplasmic domain sequences were aligned using T-Coffee (http://www.ebi.ac.uk/Tools/msa/tcoffee/) [63], both allowing gaps.

### Matlab code

A custom-built MATLAB script (MATLAB version 7.10.0.499, The Mathworks, Inc., Natick, MA) was used to identify putative PDZ ligand motifs in MHCI cytoplasmic domain sequences. PDZ ligand consensus motifs used in Fig. 1 and Additional file 2: Figure S2: class 1, [(S/T) X (Y/F/W/C/M/V/I/L/A)], where X is any amino acid; class 2, [(Y/F/W/C/M/V/I/L/A) X (Y/F/W/C/M/V/I/L/A)]; class 3, [(D/E) X (Y/F/W/C/M/V/I/L/A)] [49]. For Fig. 2a–c, PDZ ligand consensus motifs were: class 2, [X Φ X Φ], and class 3, [X (D/E) X Φ], where is Φ defined as V/I/L/M/F/W/C, and X is any amino acid. All MATLAB code generated for use in this study is freely available on the Boulanger lab website (http://www.boulangerlab.org/).

### Frequency of class 1 PDZ ligand motifs in the extracellular versus cytoplasmic domains

Amino acid sequences for 20 mouse MHCI and MHCI-like proteins (H2-K, −D, −L, −M1, −M2, −M9, −T3, −T5, −T9, −T11, −T15, −T22, −T23 (also known as Qa-1), −T24, −Q1, −Q2, −Q5, HFE, FcRn, and MR1) were obtained from the NCBI Protein Database (http://www.ncbi.nlm.nih.gov/protein). Cytoplasmic, transmembrane, and extracellular domains of each sequence were identified by prediction of transmembrane position by hydrophobicity (TMHMM; http://www.cbs.dtu.dk/services/TMHMM/) as well as by sequence homology with MHCI proteins of known topology. Class 1 PDZ ligand motifs were identified using a lab-built MATLAB program (see above), and putative PDZ ligand frequency was defined as the number of putative PDZ ligands per 100 amino acids. To generate the baseline “random” frequency of PDZ ligand motifs for a given sequence, each amino acid sequence was scrambled using an online text scrambler (http://textmechanic.com/Word-Scrambler.html), and all PDZ ligand motifs identified using MATLAB. This process was repeated ten times for each sequence to generate an estimate of the likelihood of a PDZ ligand appearing by chance, appropriately weighted by the amino acid composition of each protein. Randomization was performed for each MHCI protein individually, and the values were averaged across genes. For scrambled sequence, the full amino acid sequence of a given MHCI protein was randomized and searched for ligands or inverse ligands. The peptide was then randomized again and searched again. Ten randomization replicates per protein were performed to obtain an estimate of the average chance of randomly generating putative PDZ ligands from that particular pool of amino acids. The total number of each motif was compiled in Excel (Microsoft) and graphed in Origin (Microcal).

### Relative frequencies of specific class 1 and class 2 PDZ ligand motifs in the cytoplasmic domain

Full length amino acid sequences for 99 MHCI or MHCI-like proteins from 21 species were obtained using STRING (see above). Sequences were searched using custom-designed MATLAB software for the presence of specific forms of putative class 1 [X (S/T) X (V/L)], class 2 ([XΦXΦ], where Φ = (VILMFWC)), or class 3 [X (D/E) X Φ] PDZ ligand motifs [51]. The total number of each specific motif in the extracellular and cytoplasmic domains of all proteins was complied in Excel (Microsoft) and graphed in Origin (Microcal).

### Representation of amino acid conservation in logos

The Logos Look Amazing (LOLA) program was used to visualize amino acid sequence conservation.

LOLA was created by the Bader lab and is freely available on their lab website (http://baderlab.org/Software/LOLA) [50].

### Cloning of MHCI cytoplasmic domain and expression of recombinant peptides

All procedures were performed in accordance with protocols approved by the Princeton University Institutional Animal Care and Use Committee (IACUC). Mice were maintained on a 12 h light-dark cycle with food and water access ad libitum. C57BL/6 mice were obtained from The Jackson Laboratory (Bar Harbor, ME). Postnatal day 14 C57BL/6 mice were deeply anesthetized to a surgical plane via inhalation anesthesia, decapitated, their brains rapidly removed, and hippocampi microdissected. Tissue was homogenized via mortar and pestle and the RNA was extracted and purified using a Qiagen RNeasy Mini Kit (Valencia, CA). RT-PCR of total RNA was performed using the Quanta Biosciences qScript cDNA Synthesis Kit (Gaithersburg, MD). C-terminal sequences of the classical MHCI H2-K^b^ were amplified using PCR with the primer sequences shown in Additional file 4: Figure S4A. Restriction sites (BglII and NcoI) were included flanking each primer, allowing them to be cloned into the pET29a vector. All MHCI and PDZ constructs were commercially sequenced (GeneWiz) to verify correct insertion of the target sequence. After sequencing, constructs were subcloned into a pGTK vector (gift of T. Shenk) via BamHI and HindIII restriction sites for N-terminal GST tagging, to facilitate purification and detectection. Site-directed mutagenesis (T329A) was similarly introduced in the pET29a vector, verified by sequencing, and cloned into the pGTK vector.

GST-tagged C-terminal MHCI peptides and 6xHis-tagged PDZ domain constructs for MAGI-1 and SAP97 (gift of R. Hall) were expressed in BL21(DE3) competent *E. coli* (New England Biolabs, Ipswich, MA) using 1.2 L (MHCI peptides) or 10 ml (PDZ peptides) of Overnight Express Instant TB Media (EMD Millipore, Billerica, MA). Cell lysates were applied to columns (equilibrated glutathione columns (Pierce GST Spin Purification Kit) for GST-tagged MHCI cytoplasmic domains, or Pierce HisPur Cobalt Spin Purification columns (0.2 ml resin bed, Rockland, IL) for 6xHis-tagged PDZ domains) to bind the expressed peptides, and washed according to the manufacturer’s protocols. Recombinant proteins were desalted using Thermo Fisher desalting columns, following the manufacturer’s protocol, and quantified using a Pierce BCA Protein Assay kit.

### In vitro binding assays

Binding assays were performed as previously described [70]. Briefly, purified recombinant 6xHis-tagged PDZ domain peptides were spotted (1 μg of each peptide per spot; for titration experiments, 5 μg/spot) onto a nitrocellulose membrane and dried. Membranes were incubated in blocking buffer (2 % nonfat dry milk, 0.1 % Tween 20, 50 mM NaCl, 10 mM Hepes, pH 7.4) for 1 h at RT. Membranes were incubated in GST-tagged recombinant MHCI cytoplasmic domain peptides (200 nM, unless otherwise noted, diluted in blocking buffer) overnight at 4 °C with shaking. Membranes were washed 3×5 min in TBST and then incubated with rabbit anti-GST primary antibody (1:1000) (Thermo (8–326)) for 1 h at RT. Membranes were washed 5×5 min in TBST and incubated in HRP-conjugated anti-rabbit secondary antibody (1:5000; Rockland Immunochemicals, Gillbertsville, PA) for 1 h at RT, followed by washing in TBST. Membranes were treated with West Pico Chemiluminescent Substrate (Pierce, Rockland, IL) and exposed on HyBlot CL Autoradiography Film (Denville Scientific, Metuchen, NJ). Densitometry was performed on scanned Western blots and quantified using the ImageJ 1.32 software (National Institutes of Health, Bethesda, MD). Background measurements were made on the same blot in areas where no bands or spots were detected, and these background values were subtracted from density of the bands of interest to obtain background-subtracted values.

### Competition experiment

GST-tagged C-terminal MHCI peptides were expressed in BL21(DE3) competent *E. coli* (New England Biolabs, Ipswich, MA) using 1.2 L of Overnight Express Instant TB Media (EMD Millipore, Billerica, MA). Cell lysates were applied to equilibrated glutathione columns (Pierce GST Spin Purification Kit) for GST-tagged MHCI cytoplasmic domains. Recombinant GST proteins were desalted using Thermo Fisher desalting columns, following the manufacturer’s protocol, and quantified using a Pierce BCA Protein Assay kit. His-tagged MAGI-1 were expressed in BL21(DE3) competent *E. coli* (New England Biolabs, Ipswich, MA) using 5 mL of Overnight Express Instant TB Media (EMD Millipore, Billerica, MA). Cell lysates were bound to HisPur Cobalt Spin Purification columns (0.2 ml resin bed, Rockland, IL) and incubated overnight at 4 °C. Columns were washed 3 times according to manufacturer’s protocols and preincubated with respective competitor GST MHCI peptide (GST-H2-T22 or GST-H2-D) at a concentration of 200 nM in manufacturer’s wash buffer for 2 h at 4 °C. Following preincubation, 200 nM of GST-H2-K was added to the column for 2 h at 4 °C. Columns were washed 5 times in manufacturer’s wash buffer at 700×g for 2 min, and bound peptides were eluted according to manufacturer’s protocols (400 μL of elution buffer, overnight incubation, 4 °C). Samples were prepared as follows: 30 μL of eluate + 10 μL of 4× sample buffer were boiled for 10 min and run on a 4–20 % gradient gel (BioRad), followed by western blotting (below) to visualize proteins.

### Western blotting

To verify the identity of the recombinant GST-tagged MHCI cytoplasmic domain peptide, 1 μg of purified GST-H2-K^b^ was subjected to SDS-PAGE on a 4–20 % gradient gel (Bio-Rad) and transferred to an Immobilon-P PVDF transfer membrane (Millipore) as previously described [9]. PVDF membranes were blocked overnight at 4 °C in blocking buffer (5 % milk in TBST [20 mM Tris pH 7.5, 150 mM NaCl, 0.1 % Tween 20]). Primary antibodies (mouse anti-MHCI [p8, 1:5000, purified rabbit polyclonal raised against exon 8 of H-2K, GeneScript]) were diluted in blocking buffer and incubated with the membranes overnight at 4 °C. Membranes were washed for 12×5min in TBST, followed by a 1 h incubation at RT with HRP-conjugated goat anti-rabbit secondary antibody (1:20,000; Jackson ImmunoResearch Laboratories # 111:035:144) and washing for 12×5 min in TBST. Membranes were treated with Pierce ECL Western Blotting Substrate (#32106, Rockland, IL) and exposed on HyBlot CL Autoradiography Film (Denville Scientific, Metuchen, NJ). A single strongly-reactive band of the predicted size for the GST fusion protein (30 kDa) was detected (Additional file 4: Figure S4C).

For competition experiments, samples were prepared and run on a SDS-PAGE 4–20 % gradient gel (Bio-Rad) and transferred to a PVDF membrane (Millipore). Membranes were incubated in an antibody raised against exon 8, in the cytoplasmic domain of H2-K (P8 (GeneScript), 1:5,000 dilution in 5 % organic dry milk), followed by 12×5 min washes in TBST. Membranes were then incubated in HRP-conjugated goat anti-rabbit secondary (1:10,000 dilution) for 1 h at RT, and washed 7×5 min in TBST.

Membranes were incubated in stripping buffer (15 g glycine, 1 g SDS, 10 ml Tween-20, pH 2.2) for 15 min and washed 3×5mins in TBST. The membranes were blocked overnight at 4 ° C in 3 % BSA in TBST and re-probed with anti-HIS antibody (1:5,000 dilution in 3 % BSA; Cell Signaling Technology #2364) overnight at 4 °C. Membranes were washed 12×5min in TBST and incubated in HRP-conjugated goat anti-rabbit secondary antibody (1:10,000 dilution) for 1 h at RT and washed 7×5min in TBST. Blots were treated with ECL as above and used to expose film. The density of the bands on Western blots (below) was quantified using ImageJ software and calculated as the ratio of the density of H2-K over the density of HIS-PDZ.

### Proteomic sample preparation and MS data acquisition

We performed mass spectrometry on samples that contained high concentrations of the GST-H2-K^b^ fusion peptide or the GST-H2-K^b^ T329A point mutant peptide. Protein solutions were subjected to buffer exchange into 8 M urea, followed by thiol reduction and alkylation, and tryptic digestion using the FASP procedure [97]. Peptides were desalted using StageTip micro-scale reversed-phase C18 chromatography [98] and subjected to reversed-phase nano-LC-MS and MS/MS on an Orbitrap Elite hybrid mass spectrometer (ThermoFisher), outfitted with an Easy nLC 1000 Ultra nano-UPLC system (Proxeon) and a Flex ion source (Proxeon). Sample concentration and washing was achieved online using a trapping capillary column (150 μm × ca. 40 mm, packed with 3 μm, 100 Å Magic AQ C18 resin, Michrom, Auburn, CA) at a flow rate of 5 μL/min for ca. 5 min, while separation was achieved using an extended analytical capillary column (75 μm × ca. 45 cm, packed with 3 μm, 100 Å Magic AQ C18 resin, Michrom) under a linear gradient of A and B solutions (solution A: 3 % acetonitrile/0.1 % formic acid; solution B: 97 % acetonitrile/0.1 % formic acid) over 180 min at a flow rate of 300 nL/min with column heating at 55 °C. Nano electrospray ionization was carried out at 2.4 kV, with the Elite heated capillary set to 275 °C. Full-scan mass spectra were acquired in the Orbitrap in positive-ion mode over the m/z range of 335–1800 at a resolution of 120,000 and the top 15 most abundant multiply charged species in every full scan were subject to MS/MS. Lockmass linear recalibration was employed using a siloxane background ion (*m/z* 445), which maintained absolute mass accuracy to within 2–3 ppm.

### MS data analysis

Resultant LC-MS/MS data were searched against custom databases consisting of MHCI fusion protein construct sequences concatenated to either the UniProt Mouse proteome or a PDZ-domain-containing protein subset of the UniProt Mouse proteome, using ProteomeDiscoverer (v. 2.1, ThermoFisher Scientific) with the Mascot search engine node (v. 2.5, Matrix Science, London, UK.). Search parameters allowed for a mass error of 6 ppm for precursor species and 1.2 Da for fragments, ≤ 3 missed trypsin cleavages, methionine oxidation and *N*-terminal protein acetylation as variable modifications and carbamidomethylation of cysteines as a fixed modification. Aggregate search results for each sample were imported into Scaffold software (v. 4.4.8 Proteome Software, Portland, OR), for consolidation and visualization.

## Abbreviations

C-terminus, carboxy terminus; CTL, cytotoxic T lymphocyte; GST, glutathione S-transferase; HLA, human leukocyte antigen; HRP, horseradish peroxidase; MHCI, major histocompatibility complex class I; NCBI, National Center for Biotechnology Information; NMDA, *N*-methyl *D*-aspartate; PDZ, PSD-95/discs large/zonula occludens-1; PVDF, polyvinylidene fluoride; RT, room temperature; SDS-PAGE, sodium dodecyl sulfate polyacrylamide gel electrophoresis; TBS, tris-buffered saline; TBST, tris-buffered saline + Tween 20

## Additional files

Additional file 1: Figure S1. Categorization of PDZ ligands. The current study made use of forms of Lenfant et al.’s [44] and Nourry et al.’s [46] criteria to identify potential PDZ ligand motifs in MHCI proteins. Related rules were proposed in [45]. (PPTX 35 kb) Additional file 2: Figure S2. PDZ ligand motifs identified in the cytoplasmic domains of 16 mouse MHCI and MHCI-like proteins. Putative PDZ ligand motifs are highlighted in red, and previously noted conserved serines and tyrosine (see text) are underlined in bold. Consensus motifs: class 1 PDZ, [S/T X Φ]; class 2 PDZ, [ΦXΦ]; class 3 PDZ, [D/E X Φ]; Φ = Y, F, W, C, M, V, I, L, or A [44]. H2-T23 is also known as Qa-1. No ligand motifs were found in the cytoplasmic domain of H2-M2 or -M9, and therefore they are omitted. Soluble MHCI proteins including H2-Q10 lack a cytoplasmic domain and were not considered in this analysis. (PPTX 37 kb) Additional file 3: Figure S3. Frequency of occurrence of specific class 2 and 3 PDZ ligand motifs in the cytoplasmic domains of 99 MHCI proteins from 21 species. **A.** Class 2 PDZ ligand motifs ([ΦXΦ], where Φ = V, I, L, M, F, W, or C), or **B.** class 3 PDZ ligand motifs ([D/E X Φ) [46]) in the cytoplasmic domains of 99 MHCI proteins from 21 species (data used to create pie charts in Fig. 2e and f, respectively). Shown are the number of occurrences of each motif as well as the fraction they represent of the total motifs observed in each domain. Class 2 PDZ ligand forms that were never observed in either domain are not shown. (PPTX 73 kb) Additional file 4: Figure S4. Molecular validation of tools used in Fig. 3. **A.** Sequences of primers used to clone native MHCI cytoplasmic domains from whole mouse brain, as well as primers used to generate point mutations and deletions in cloned sequences. pET29a primers were used to amplify target sequences and introduce point mutations; pGTK primers were used for subcloning into a vector for N-terminal GST tagging and subsequent sequencing, purification and detection. **B-C.** Confirmation of the amino acid sequence of recombinant H2-K cytoplasmic peptides employed in binding experiments by MS. Peptide spectral matches to (**B**) H2-K and (**C**) the T329A mutant of H2-K sequences found in the LC-MS/MS data. Prominent fragment ions are labeled with their b-/y- type ion designations. Inset into the spectra (above) are sequence fragmentation flag diagrams summarizing all the detected fragment ions present in the spectra. The detected mutation sites are highlighted in the sequences in red. **D.** Recombinant GST-tagged H2-K peptide is recognized by an antibody raised against exon 8 of the cytoplasmic domain of H2-K. The immunoreactive band (arrow) runs at the predicted molecular weight for GST-H2-K cytoplasmic domain (30 kDa). Numbers, molecular weight in kDa. **E.-F.** Titration experiments with increasing concentrations of H2-K cytoplasmic domain peptides and constant concentrations of MAGI-1-derived peptides. H2-K cytoplasmic domain binds to MAGI-1 PDZ1 and PDZ3 in a dose-dependent manner. Bottom row, H2-K peptide spotted directly on the membrane (positive control). Controls for in vitro binding specificity show that GST alone shows no specific binding to any PDZ domain from MAGI-1. **F**, Densitometry-based quantification of H2-K binding to MAGI-1 PDZ1 for the experiment shown in **E**. (PPTX 1995 kb)
