# Supplementary material for: Cryptic protein-protein interaction motifs in the cytoplasmic domain of MHCI proteins
Source: BMC Immunol. 2016 Jul 19;17:24. doi: 10.1186/s12865-016-0154-z (PMC4950430; doi:10.1186/s12865-016-0154-z)
Supplement: Additional file 1: Figure S1. — Categorization of PDZ ligands. The current study made use of forms of Lenfant et al.’s [44] and Nourry et al.’s [46] criteria to identify potential PDZ ligand motifs in MHCI proteins. Related rules were proposed in [45]. (PPTX 35 kb) [file 12865_2016_154_MOESM1_ESM.pptx]

## Slide 1
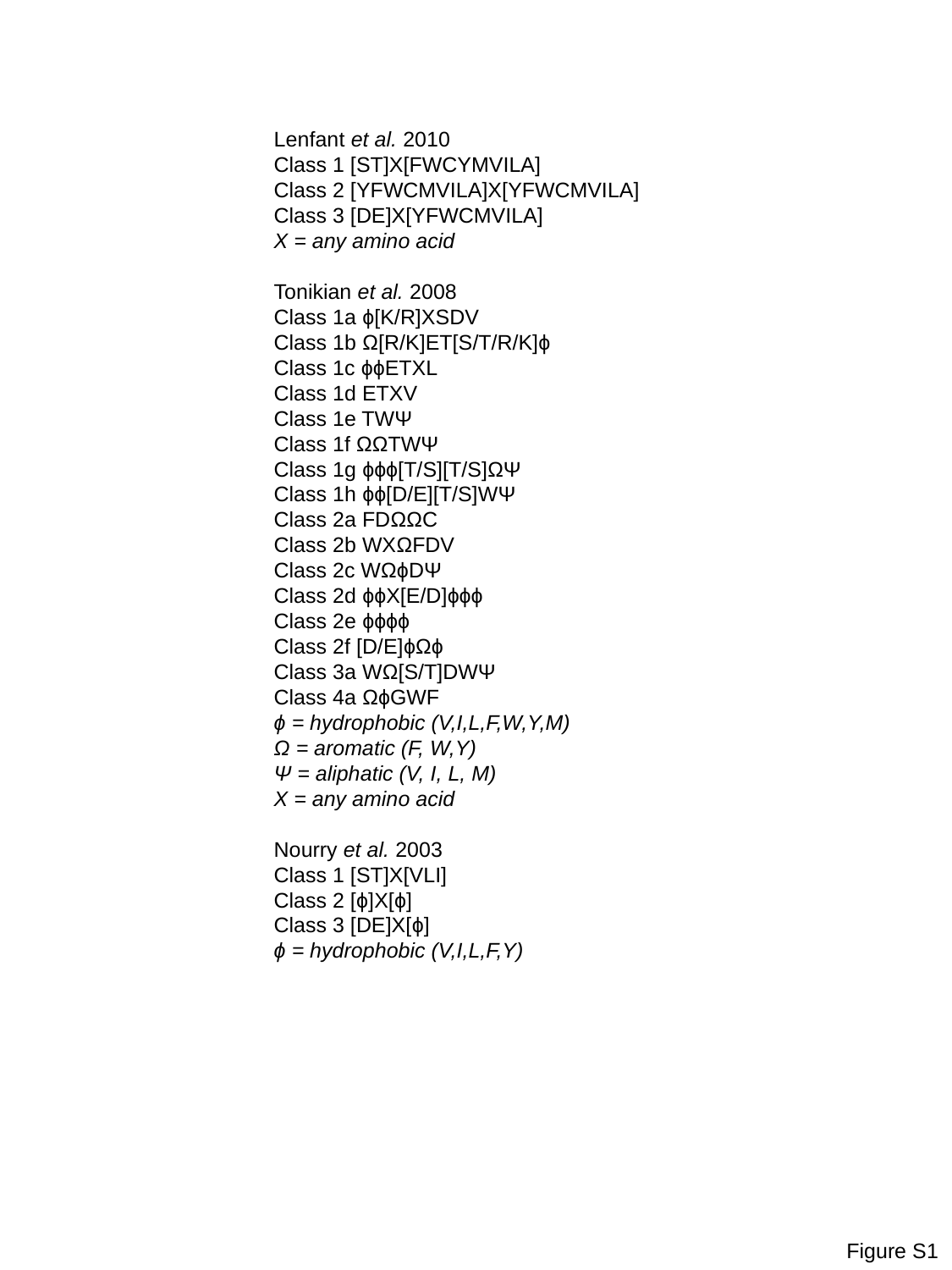

Lenfant et al. 2010
Class 1 [ST]X[FWCYMVILA]
Class 2 [YFWCMVILA]X[YFWCMVILA]
Class 3 [DE]X[YFWCMVILA]
X = any amino acid
Tonikian et al. 2008
Class 1a ϕ[K/R]XSDV
Class 1b Ω[R/K]ET[S/T/R/K]ϕ
Class 1c ϕϕETXL
Class 1d ETXV
Class 1e TWΨ
Class 1f ΩΩTWΨ
Class 1g ϕϕϕ[T/S][T/S]ΩΨ
Class 1h ϕϕ[D/E][T/S]WΨ
Class 2a FDΩΩC
Class 2b WXΩFDV
Class 2c WΩϕDΨ
Class 2d ϕϕX[E/D]ϕϕϕ
Class 2e ϕϕϕϕ
Class 2f [D/E]ϕΩϕ
Class 3a WΩ[S/T]DWΨ
Class 4a ΩϕGWF
ϕ = hydrophobic (V,I,L,F,W,Y,M)
Ω = aromatic (F, W,Y)
Ψ = aliphatic (V, I, L, M)
X = any amino acid
Nourry et al. 2003
Class 1 [ST]X[VLI]
Class 2 [ϕ]X[ϕ]
Class 3 [DE]X[ϕ]
ϕ = hydrophobic (V,I,L,F,Y)
Figure S1
