# Supplementary material for: Cryptic protein-protein interaction motifs in the cytoplasmic domain of MHCI proteins
Source: BMC Immunol. 2016 Jul 19;17:24. doi: 10.1186/s12865-016-0154-z (PMC4950430; doi:10.1186/s12865-016-0154-z)
Supplement: Additional file 2: Figure S2. — PDZ ligand motifs identified in the cytoplasmic domains of 16 mouse MHCI and MHCI-like proteins. Putative PDZ ligand motifs are highlighted in red, and previously noted conserved serines and tyrosine (see text) are underlined in bold. Consensus motifs: class 1 PDZ, [S/T X Φ]; class 2 PDZ, [ΦXΦ]; class 3 PDZ, [D/E X Φ]; Φ = Y, F, W, C, M, V, I, L, or A [44]. H2-T23 is also known as Qa-1. No ligand motifs were found in the cytoplasmic domain of H2-M2 or -M9, and therefore they are omitted. Soluble MHCI proteins including H2-Q10 lack a cytoplasmic domain and were not considered in this analysis. (PPTX 37 kb) [file 12865_2016_154_MOESM2_ESM.pptx]

## Slide 1
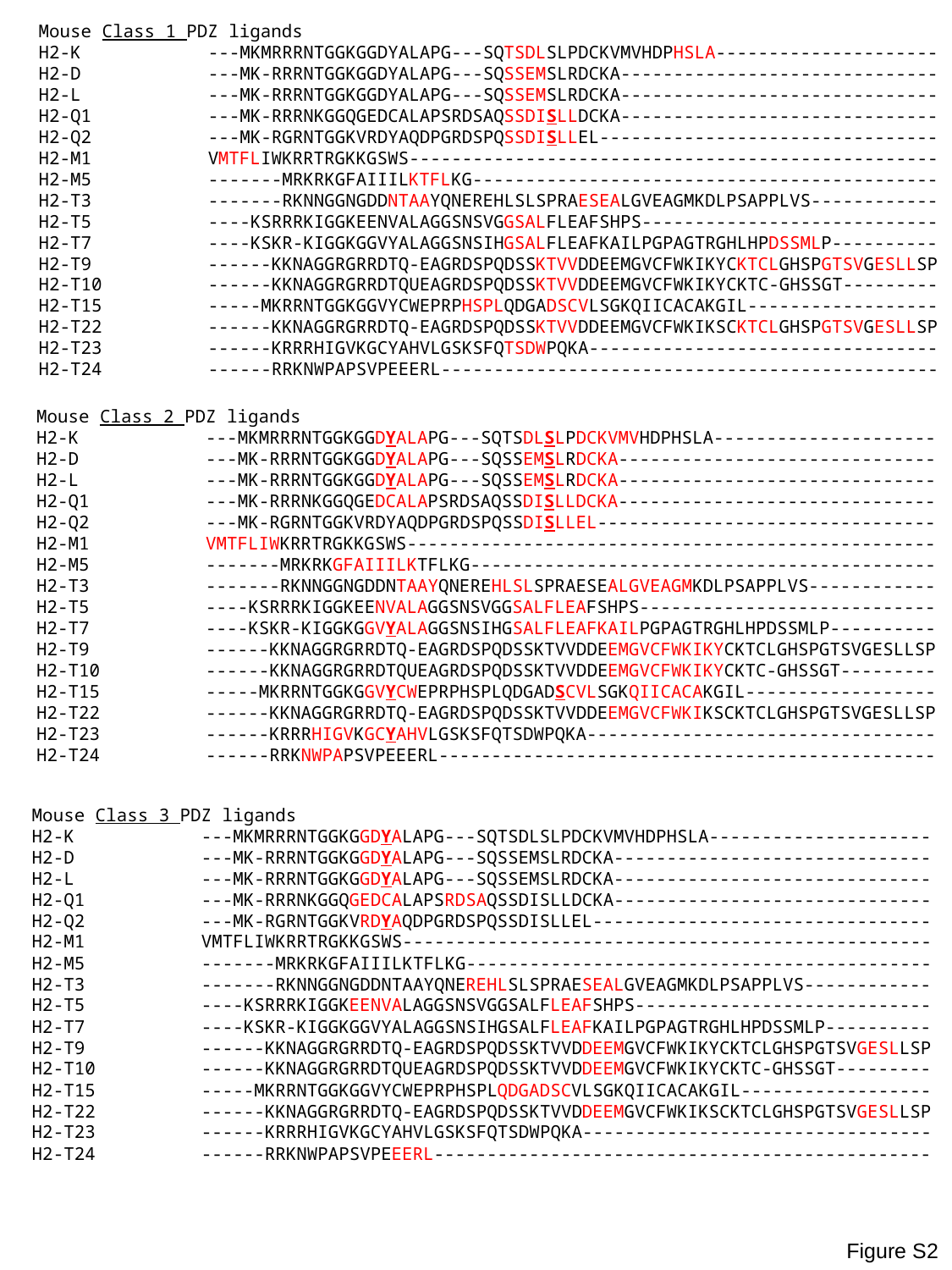

Mouse Class 1 PDZ ligands
H2-K ---MKMRRRNTGGKGGDYALAPG---SQTSDLSLPDCKVMVHDPHSLA---------------------
H2-D ---MK-RRRNTGGKGGDYALAPG---SQSSEMSLRDCKA------------------------------
H2-L ---MK-RRRNTGGKGGDYALAPG---SQSSEMSLRDCKA------------------------------
H2-Q1 ---MK-RRRNKGGQGEDCALAPSRDSAQSSDISLLDCKA------------------------------
H2-Q2 ---MK-RGRNTGGKVRDYAQDPGRDSPQSSDISLLEL--------------------------------
H2-M1 VMTFLIWKRRTRGKKGSWS--------------------------------------------------
H2-M5 -------MRKRKGFAIIILKTFLKG--------------------------------------------
H2-T3 -------RKNNGGNGDDNTAAYQNEREHLSLSPRAESEALGVEAGMKDLPSAPPLVS------------
H2-T5 ----KSRRRKIGGKEENVALAGGSNSVGGSALFLEAFSHPS----------------------------
H2-T7 ----KSKR-KIGGKGGVYALAGGSNSIHGSALFLEAFKAILPGPAGTRGHLHPDSSMLP----------
H2-T9 	 ------KKNAGGRGRRDTQ-EAGRDSPQDSSKTVVDDEEMGVCFWKIKYCKTCLGHSPGTSVGESLLSP
H2-T10 ------KKNAGGRGRRDTQUEAGRDSPQDSSKTVVDDEEMGVCFWKIKYCKTC-GHSSGT---------
H2-T15 -----MKRRNTGGKGGVYCWEPRPHSPLQDGADSCVLSGKQIICACAKGIL------------------
H2-T22 	 ------KKNAGGRGRRDTQ-EAGRDSPQDSSKTVVDDEEMGVCFWKIKSCKTCLGHSPGTSVGESLLSP
H2-T23 	 ------KRRRHIGVKGCYAHVLGSKSFQTSDWPQKA---------------------------------
H2-T24 ------RRKNWPAPSVPEEERL-----------------------------------------------
Mouse Class 2 PDZ ligands
H2-K ---MKMRRRNTGGKGGDYALAPG---SQTSDLSLPDCKVMVHDPHSLA---------------------
H2-D ---MK-RRRNTGGKGGDYALAPG---SQSSEMSLRDCKA------------------------------
H2-L ---MK-RRRNTGGKGGDYALAPG---SQSSEMSLRDCKA------------------------------
H2-Q1 ---MK-RRRNKGGQGEDCALAPSRDSAQSSDISLLDCKA------------------------------
H2-Q2 ---MK-RGRNTGGKVRDYAQDPGRDSPQSSDISLLEL--------------------------------
H2-M1 VMTFLIWKRRTRGKKGSWS--------------------------------------------------
H2-M5 -------MRKRKGFAIIILKTFLKG--------------------------------------------
H2-T3 -------RKNNGGNGDDNTAAYQNEREHLSLSPRAESEALGVEAGMKDLPSAPPLVS------------
H2-T5 ----KSRRRKIGGKEENVALAGGSNSVGGSALFLEAFSHPS----------------------------
H2-T7 ----KSKR-KIGGKGGVYALAGGSNSIHGSALFLEAFKAILPGPAGTRGHLHPDSSMLP----------
H2-T9 	 ------KKNAGGRGRRDTQ-EAGRDSPQDSSKTVVDDEEMGVCFWKIKYCKTCLGHSPGTSVGESLLSP
H2-T10 ------KKNAGGRGRRDTQUEAGRDSPQDSSKTVVDDEEMGVCFWKIKYCKTC-GHSSGT---------
H2-T15 -----MKRRNTGGKGGVYCWEPRPHSPLQDGADSCVLSGKQIICACAKGIL------------------
H2-T22 	 ------KKNAGGRGRRDTQ-EAGRDSPQDSSKTVVDDEEMGVCFWKIKSCKTCLGHSPGTSVGESLLSP
H2-T23 	 ------KRRRHIGVKGCYAHVLGSKSFQTSDWPQKA---------------------------------
H2-T24 ------RRKNWPAPSVPEEERL-----------------------------------------------
Mouse Class 3 PDZ ligands
H2-K ---MKMRRRNTGGKGGDYALAPG---SQTSDLSLPDCKVMVHDPHSLA---------------------
H2-D ---MK-RRRNTGGKGGDYALAPG---SQSSEMSLRDCKA------------------------------
H2-L ---MK-RRRNTGGKGGDYALAPG---SQSSEMSLRDCKA------------------------------
H2-Q1 ---MK-RRRNKGGQGEDCALAPSRDSAQSSDISLLDCKA------------------------------
H2-Q2 ---MK-RGRNTGGKVRDYAQDPGRDSPQSSDISLLEL--------------------------------
H2-M1 VMTFLIWKRRTRGKKGSWS--------------------------------------------------
H2-M5 -------MRKRKGFAIIILKTFLKG--------------------------------------------
H2-T3 -------RKNNGGNGDDNTAAYQNEREHLSLSPRAESEALGVEAGMKDLPSAPPLVS------------
H2-T5 ----KSRRRKIGGKEENVALAGGSNSVGGSALFLEAFSHPS----------------------------
H2-T7 ----KSKR-KIGGKGGVYALAGGSNSIHGSALFLEAFKAILPGPAGTRGHLHPDSSMLP----------
H2-T9 	 ------KKNAGGRGRRDTQ-EAGRDSPQDSSKTVVDDEEMGVCFWKIKYCKTCLGHSPGTSVGESLLSP
H2-T10 ------KKNAGGRGRRDTQUEAGRDSPQDSSKTVVDDEEMGVCFWKIKYCKTC-GHSSGT---------
H2-T15 -----MKRRNTGGKGGVYCWEPRPHSPLQDGADSCVLSGKQIICACAKGIL------------------
H2-T22 	 ------KKNAGGRGRRDTQ-EAGRDSPQDSSKTVVDDEEMGVCFWKIKSCKTCLGHSPGTSVGESLLSP
H2-T23 	 ------KRRRHIGVKGCYAHVLGSKSFQTSDWPQKA---------------------------------
H2-T24 ------RRKNWPAPSVPEEERL-----------------------------------------------
Figure S2
