# Supplementary figures and images for: Cryptic protein-protein interaction motifs in the cytoplasmic domain of MHCI proteins
Source: BMC Immunol. 2016 Jul 19;17:24. doi: 10.1186/s12865-016-0154-z (PMC4950430; doi:10.1186/s12865-016-0154-z)

## Slide 1
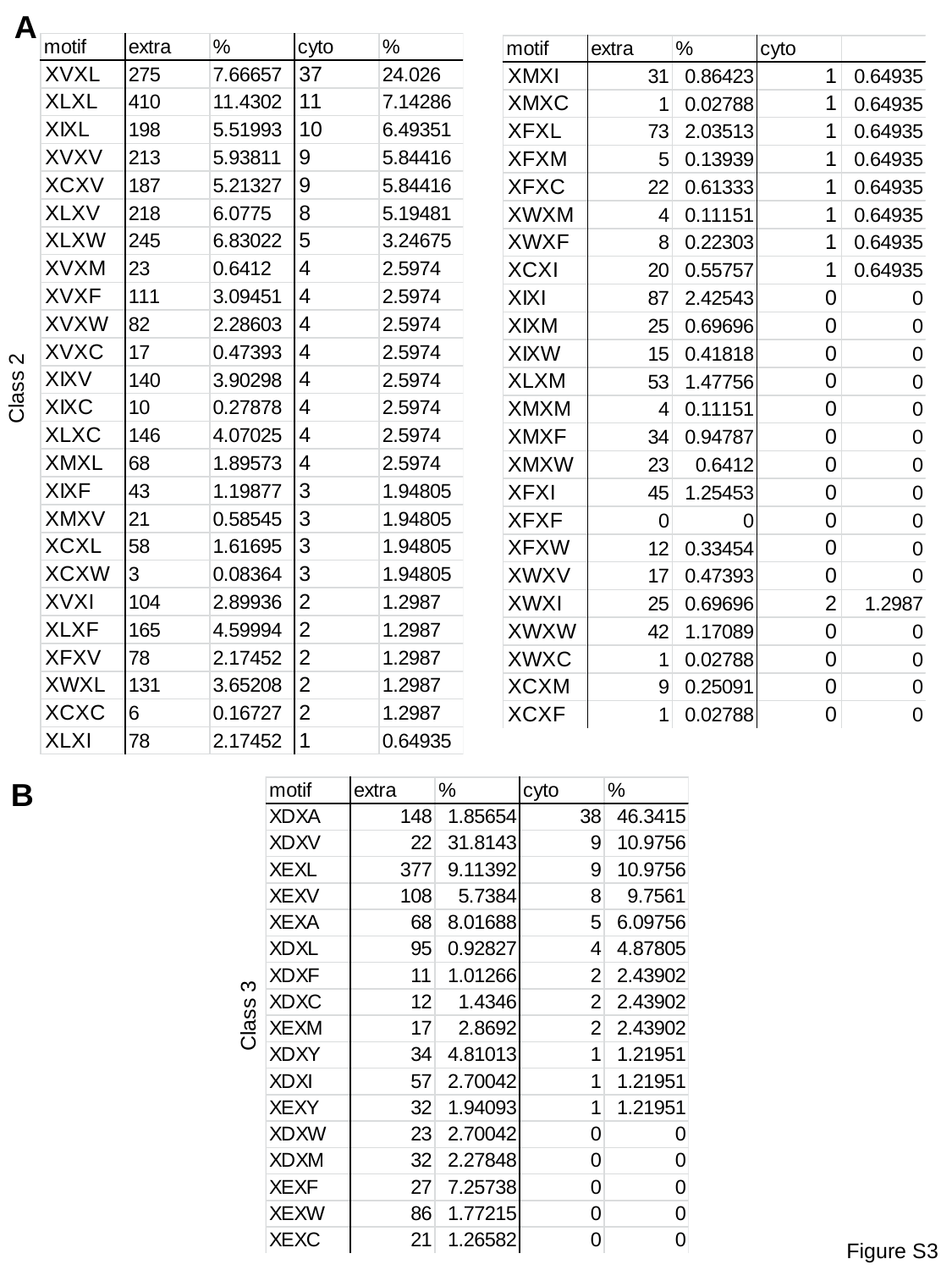

A
Class 2
B
Class 3
Figure S3

Supplement: Additional file 3: Figure S3. — Frequency of occurrence of specific class 2 and 3 PDZ ligand motifs in the cytoplasmic domains of 99 MHCI proteins from 21 species. A. Class 2 PDZ ligand motifs ([ΦXΦ], where Φ = V, I, L, M, F, W, or C), or B. class 3 PDZ ligand motifs ([D/E X Φ) [46]) in the cytoplasmic domains of 99 MHCI proteins from 21 species (data used to create pie charts in Fig. 2e and f, respectively). Shown are the number of occurrences of each motif as well as the fraction they represent of the total motifs observed in each domain. Class 2 PDZ ligand forms that were never observed in either domain are not shown. (PPTX 73 kb) [file 12865_2016_154_MOESM3_ESM.pptx]
