# Supplementary material for: Cryptic protein-protein interaction motifs in the cytoplasmic domain of MHCI proteins
Source: BMC Immunol. 2016 Jul 19;17:24. doi: 10.1186/s12865-016-0154-z (PMC4950430; doi:10.1186/s12865-016-0154-z)
Supplement: Additional file 4: Figure S4. — Molecular validation of tools used in Fig. 3. A. Sequences of primers used to clone native MHCI cytoplasmic domains from whole mouse brain, as well as primers used to generate point mutations and deletions in cloned sequences. pET29a primers were used to amplify target sequences and introduce point mutations; pGTK primers were used for subcloning into a vector for N-terminal GST tagging and subsequent sequencing, purification and detection. B-C. Confirmation of the amino acid sequence of recombinant H2-K cytoplasmic peptides employed in binding experiments by MS. Peptide spectral matches to (B) H2-K and (C) the T329A mutant of H2-K sequences found in the LC-MS/MS data. Prominent fragment ions are labeled with their b-/y- type ion designations. Inset into the spectra (above) are sequence fragmentation flag diagrams summarizing all the detected fragment ions present in the spectra. The detected mutation sites are highlighted in the sequences in red. D. Recombinant GST-tagged H2-K peptide is recognized by an antibody raised against exon 8 of the cytoplasmic domain of H2-K. The immunoreactive band (arrow) runs at the predicted molecular weight for GST-H2-K cytoplasmic domain (30 kDa). Numbers, molecular weight in kDa. E.-F. Titration experiments with increasing concentrations of H2-K cytoplasmic domain peptides and constant concentrations of MAGI-1-derived peptides. H2-K cytoplasmic domain binds to MAGI-1 PDZ1 and PDZ3 in a dose-dependent manner. Bottom row, H2-K peptide spotted directly on the membrane (positive control). Controls for in vitro binding specificity show that GST alone shows no specific binding to any PDZ domain from MAGI-1. F, Densitometry-based quantification of H2-K binding to MAGI-1 PDZ1 for the experiment shown in E. (PPTX 1995 kb) [file 12865_2016_154_MOESM4_ESM.pptx]

## Slide 1
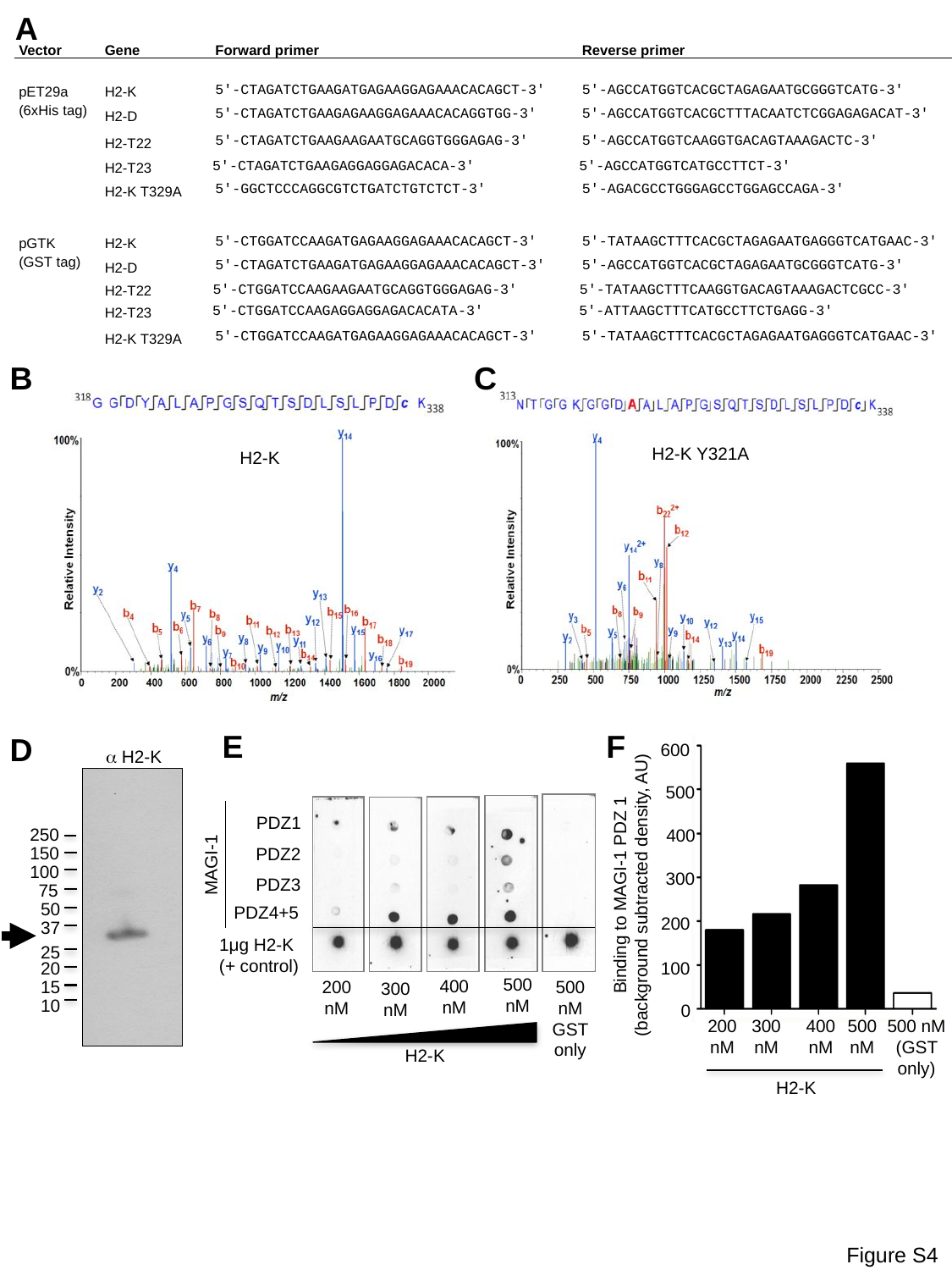

A
| Vector | Gene | Forward primer | Reverse primer |
| --- | --- | --- | --- |
| | | | |
| pET29a (6xHis tag) | H2-K | 5'-CTAGATCTGAAGATGAGAAGGAGAAACACAGCT-3' | 5'-AGCCATGGTCACGCTAGAGAATGCGGGTCATG-3' |
| | H2-D | 5'-CTAGATCTGAAGAGAAGGAGAAACACAGGTGG-3' | 5'-AGCCATGGTCACGCTTTACAATCTCGGAGAGACAT-3' |
| | H2-T22 | 5'-CTAGATCTGAAGAAGAATGCAGGTGGGAGAG-3' | 5'-AGCCATGGTCAAGGTGACAGTAAAGACTC-3' |
| | H2-T23 | 5'-CTAGATCTGAAGAGGAGGAGACACA-3' | 5'-AGCCATGGTCATGCCTTCT-3' |
| | H2-K T329A | 5'-GGCTCCCAGGCGTCTGATCTGTCTCT-3' | 5'-AGACGCCTGGGAGCCTGGAGCCAGA-3' |
| | | | |
| pGTK (GST tag) | H2-K | 5'-CTGGATCCAAGATGAGAAGGAGAAACACAGCT-3' | 5'-TATAAGCTTTCACGCTAGAGAATGAGGGTCATGAAC-3' |
| | H2-D | 5'-CTAGATCTGAAGATGAGAAGGAGAAACACAGCT-3' | 5'-AGCCATGGTCACGCTAGAGAATGCGGGTCATG-3' |
| | H2-T22 | 5'-CTGGATCCAAGAAGAATGCAGGTGGGAGAG-3' | 5'-TATAAGCTTTCAAGGTGACAGTAAAGACTCGCC-3' |
| | H2-T23 | 5'-CTGGATCCAAGAGGAGGAGACACATA-3' | 5'-ATTAAGCTTTCATGCCTTCTGAGG-3' |
| | H2-K T329A | 5'-CTGGATCCAAGATGAGAAGGAGAAACACAGCT-3' | 5'-TATAAGCTTTCACGCTAGAGAATGAGGGTCATGAAC-3' |
C
B
H2-K
H2-K Y321A
E
F
D
600
500
400
Binding to MAGI-1 PDZ 1
(background subtracted density, AU)
300
200
100
0
200 nM
300 nM
400 nM
500
nM
500 nM
(GST only)
H2-K
a H2-K
250
150
100
75
50
37
25
20
15
10
PDZ1
PDZ2
MAGI-1
PDZ3
PDZ4+5
1μg H2-K
(+ control)
500 nM
400 nM
500 nM
GST only
200 nM
300 nM
H2-K
Figure S4
